# Prophylactic efficacy of Quercetin in ameliorating the hypoxia induced vascular leakage in lungs of rats

**DOI:** 10.1371/journal.pone.0219075

**Published:** 2019-06-28

**Authors:** Ankit Tripathi, Bhuvnesh Kumar, Sarada S. K. Sagi

**Affiliations:** Nutrition Division, Defence Institute of Physiology and Allied Sciences, Timarpur, Delhi, India; National Institutes of Health, UNITED STATES

## Abstract

The objective of the study was to find out the prophylactic efficacy of Quercetin in ameliorating the hypoxia induced vascular leakage in lungs of rats. Male SD rats received different doses of quercetin @ 25mg, 50mg, 100mg and 200mg/Kg BW, 1h prior to hypobaric hypoxia exposure (7,620m, for 6h). Quercetin 50 mg/kg BW supplemented orally 1h prior to hypoxia exposure was considered to be the optimum dose, due to significant reduction (p<0.001) in lung water content and lung transvascular leakage compared to control (hypoxia, 6h). Further, biochemical analysis (ROS, MDA, GSH, GPx, LDH, and albumin) and differential expressions of proteins (IKK-α/β, NFĸB, Nrf-2,TNF-α, ICAM-1, VCAM, P-selectin, Hif-1α, VEGF, TNF-α, TGF-β, INF-γ and IL-4) were determined by western blotting and ELISA. Changes in lung parenchyma were assessed by histopathology. Quercetin (50 mg/kg BW) prophylaxis under hypoxia showed significant reduction in oxidative stress (ROS and MDA), concomitant increase in antioxidants (GSH, GPx and SOD) followed by decreased LDH and albumin extravasation in BAL fluid over hypoxia. Quercetin prophylaxis significantly down regulated hypoxia induced increase in IKKα/β and NFĸB expressions leading to reduction in the levels of pro-inflammatory cytokines (TNF-α and INF-γ) followed by up regulation of anti-inflammatory cytokines (IL-4 and INF-γ) in lungs. Further, hypoxia mediated increase in HIF-1α was stabilized and VEGF levels in lungs were significantly down regulated by quercetin supplementation, leading to reduction in vascular leakage in lungs of rats under hypoxia. However, Quercetin has also enacted as Nrf-2 activator which significantly boosted up the synthesis of GSH under hypoxic condition compared to hypoxia. Histopathological observations further confirmed that quercetin preconditioning has an inhibitory effect on progression of oxidative stress and inflammation via attenuation of NFκB and stabilization HIF-1α in lungs of rats under hypoxia.These studies indicated that quercetin prophylaxis abrogates the possibility of hypobaric hypoxia induced pulmonary edema in rats.

## 1. Introduction

High Altitude Pulmonary Edema (HAPE) is a non-cardiogenic and potentially fatal form of pulmonary edema, which is caused normally to an un-acclimatized, healthy individual due to rapid ascend to an altitude of 2,500m or above depending on the altitude, speed, the mode of ascent and most importantly the individual’s susceptibility [1]. For the first time in 1930 Alberto hurtado independently introduced the term HAPE in the medical literature and elucidated this new malady in the book entitled with Physiological and Pathological Aspects of Life at High Altitudes (1937) [2]. However, its non-cardiogenic behaviour was explained by Herbert Hultgren later in 1960 [3]. In the same year (1960), Houston brought the condition into the prominence of English speaking audience [4]. At early stages, HAPE can be diagnosed with dyspnoea with minimal efforts, non-productive cough, tightness in chest and reduced exercise performance, which in later severe stages can lead to debilitating degree of dyspnoea, productive cough with pink frothy sputum, cyanosis, tachycardia, tachypnoea and could even results into death [1,5].

Inauen et.al., (1990) and Huey et.al., (2002) have reported that HAPE occurs due to the leakage of plasma protein exudates from pulmonary vascular bed to alveolar airspaces was mainly because of elevated pulmonary artery pressure [6,7]. Similarly, studycarried out by Paralikar et.al., in 2012 has also reported that, from the micro-circulation, solutes were leaked in to the lungs when rats were exposed to acute hypoxia hypoxia[8].Apart from exaggerated pulmonary artery pressure, oxidant injury was also reported to be a factor responsible for transvascular flux in hypoxia exposed animals. Several studies revealed that, exposure to hypobaric hypoxia (HH) results into an up surge in the levels of nuclear factor kappa B (NF-κB) and increased expression of hypoxia inducible factor (Hif-1α), leading to the activation of numerous genes involved in the progression of oxidative stress, inflammation, angiogenesis, erythropoesis, apoptosis etc. [9,10].This cumulative effect would result into increased inflammation, elevated vascular leakage and ends up with the development of pulmonary edema [11,12]. The study performed by Li et.al., (2010) on a group of around 400 mountaineers was found to develop HAPE shortly after the ascent at high altitude. The possible approach to avoid or escape this condition is to bring the patient immediately to low altitudeand if immediate descent is not possible then provide oxygen inhalation or keep the person in portable hyperbaric chamber [13]. In addition to this, the administration of drugs such as- aminophylline, nifedipine, dexamethasone and acetazolamide were also considered as recommended pharmacotherapies for HAPE prevention [13,14]. However, the direct exposure to these drugs have been associated with the occurrence of several side-effects such as- aminophylline intake, can lead to vague chest discomfort and cardiotoxicity developed due to myocardial enzyme elevation[15].Nifedipine causes headache, nausea or dizziness and may sometimes leads to low blood pressure, whereas dexamethasone has been reported with the mood swings and hyperglycemia [15,16]. Similarly, the Acetazolamide treatment has also reported to be associated with the carbonic anhydrase inhibition leading to respiratory failure [17].

Based on the above stated facts concerned with HAPE occurrence, the present study has been programmed to minimize the inflammation and oxidative stress induced vascular leakage by prophylactic administration of a phytoflavonol (Quercetin). Quercetin (Quer; 3’,3’,4’,5,7-pentahydroxyflavone) is a naturally occurring dietary flavonol which comprises of two benzene rings bonded by a heterocyclic pyrone or pyran ring and is an essential flavonoids of human diet [18,19]. It is distributed widely in edible parts of all the plant products such as- roots, bulbs, tubers, leafy vegetables, fruits, tea and cocoa [20]. This flavonoid was found to be a potent anti-oxidant, anti-inflammatory, anti-blood coagulating, anti-tumoral, anti-apoptotic and an anti-aging biomolecule [21,22]. Recent reports have also claimed the coagnitive enhancing and neuroprotective roles of quercetin in ameliorating the hypobaric hypoxia(HH) induced neurodegeneration and memory impairement because of its effective anti-oxidant nature [23,24]. Moreover, quercetin pre-conditioning in both *in vitro* (B. subtilis strains) and *in vivo* (rats and mice) systems have elicited its anti-mutagenic and anti-carcinogenic nature [20].

Therefore, the present study was aimed to find out the prophylactic efficacy of quercetin in (1) attenuating the oxidative stress (2) dampening the inflammation (2) reducing the expression of pro-inflammatory cytokines(3) up regulating the levels of anti-inflammatory cytokinesand (4) reducing the transvascular leakage, there by altogether leadingto minimize the fluid flux in to the lungs of rats under hypoxia.

## 2. Materials and methods

### 2.1. Chemicals and reagents

Quercetin (3’,3’5’,7-pentoxyflavone) was procured from sigma Aldrich (St. Louis MO, USA), Dimethylsulphoxide (DMSO) from Sisco Research Laboratory (SRL, Maharashtra), 5’5’-dithio-bis-(2-nitro-benzoic acid) (DTNB) from Sigma Aldrich, Thiobarbituric acid(TBA)/Tricarboxylic acid (TCA) from SRL and Flourscein sodium salt from sigma Aldrich. All the other chemicals and reagents were of analytical grade.

### 2.2. Drug preparation

Quercetin was prepared freshly by dissolving in vehicle (0.5% DMSO) and administered orally to the animals 1h prior to the hypoxia exposure.

### 2.3. Safety profile of quercetin

No long-term detrimental effects from the use of Quercetin are noted in the literature till date[25]. Quercetin is well tolerated when used aptly. Recent studies have found that oral supplementation of Quercetin is the safest most route of administration compared to intravenous administration in animals[26]. The LD50 value of Quercetin when fed orally to the rats was reported to be 161 mg/Kg BW[27]

Quercetin uptake upto 4 g in a single dose in humans was reported to have no side-effects[28]. Concerning the toxicity of Quercetin uptake, recent reports have revealed that the dosage ranging from 2000 mg to 5000 mg per day did not show any evidence of toxicity on human health[29]. However, the Quercetin dosage ranging from 20–600 mg/kg BW in rats was reported to be safe and devoid of any toxicity[30]. In the present study, we have administered different concentrations of Quercetin to rats from 25–200 mg/kg BW and exposed them to 7,620 m for 6h. Since these rats were exposed to hypobaric hypoxia for 6h after the Quercetin prophylaxis, we did not find any adverse effect in any of the dosage groups tested.

### 2.4.Experimental animals and ethical guidelines

Male Sprague Dawley (SD) rats weighing between 180-200g were procured from the central animal facility of DIPAS-DRDO, Delhi, India. Animals were housed in experimentally designed polypropylene cages of 32*in*.×24*in*.×16*in*. dimension provided with standard conditions (25±2°C temperature, 55±5% relative humidity and 12h light/dark cycle) and free retrieval to standard laboratory food and water *ad libitum*. All protocols involving animal studies were reviewed and approved by the Institutional Animal Ethics Committee (IAEC), DIPAS, Delhi, India, accredited to Committee for the Purpose of Control and Supervision of Experiments on Animals (CPCSEA), Government of India. We have followed the standards set forth in the guide for the Care and Use of Laboratory Animals (National Academy of Sciences, Washington, DC)

### 2.5. Experimental protocol

The experiments were carried out in two phases:

Phase 1: Phase 1 studies were carried out to determine the optimal dosage of quercetin required to minimize the hypoxia induced vascular leakage from the lungs of rats exposed to hypobaric hypoxia. A dose dependent study of quercetin was carried out using 36 rats divided into 6 groups (n = 6), which were treated with different doses of quercetin viz: 25, 50, 100 and 200mg/kg BW.

Phase 2: The results of the phase 1 study has confirmedthat, the efficient minimization of transvascular leakage into lungs of rats exposed to hypoxia was obtained withquercetin dose 50mg/kg BW as compared to other doses tested. Therefore the remaining study was carried out using 50 mg/kg BW of quercetin as an optimal dose.

Phase 2 study was performed on 24 healthy, male SD rats which were categorized into 4 groups and each group comprises of 6 animals. Where, group 1 considered as normoxia control (0h) received only vehicle, group 2 animals were primarily fed with vehicle and then exposed to hypoxia (6h). Group 2 animals were considered as hypoxia control (6h). Group 3 animals were supplemented with quercetin (50mg/kg BW) without exposure and group 4 animals supplemented with quercetin (50mg/kg BW) 1h prior to hypoxia exposure (6h).

### 2.6. Exposure to hypoxia

Animals were exposed to a simulated hypobaric hypoxia chamber (Matrix, India) for 6h at an altitude of 7,620m (280mm Hg) with sustained temperature of 25±2°C. This 6h of hypoxia exposure has been opted based on previous studies by our lab demonstrating the elevated transvascular leakage at 6h of hypoxia exposure time at 7620 m [9]. Fresh air was flushed at the rate of 4l/h along with the relative humidity of 55±5% inside the hypoxia chamber. Furthermore, the partial pressure of oxygen (PO_2_) in control rats was observed to be 95±2 mm Hg whereas, in hypoxic rat PO_2_ was 36±2mm Hg, indicating that the rats were exposed to low barometric pressure at high altitude. The animals were provided with food and water *ad libitum* during hypoxia exposure. Utmost care was taken to minimize animal sufferings while performing the experiments.

### 2.7. Biochemical parameters

#### 2.7.1. Method of sacrifice

After 6h of hypoxia exposure, rats were sacrificed using Ketamine hydrochloride (80mg/kg BW) and Xylazine (20 mg/kg BW) as an anesthesia.

#### 2.7.2. Sample preparation

Normoxia and hypoxia exposed animal lungs were perfused with cold 1X PBS, washed with saline (0.9% NaCl) and homogenized (10%) using 0.154 M KCL containing PMSF, DTT and protease inhibitor cocktail (PIC) for carrying out the biochemical estimations.

#### 2.7.3. Measurement of oxidative stress

The quantification of ROS production in lungs of the rats exposed to hypoxia (6h) was determined using 2,7- dichlorofluorescein diacetate (DCFH-DA) assay. An assay mixture of tissue homogenate, potassium dihydrogen phosphate buffer and DCFH-DA was prepared. It was kept for 15 min. of incubation at room temperature (RT) and the fluorescence emitted due to the oxidation of DCFH-DA into DCF was measured spectrofluorimetrically (Synergy H1, Biotek, Germany) at an excitation of 485nm and emission of 530nm [31].

#### 2.7.4. Malondialdehyde estimation (MDA)

Thiobarbituric Acid Reactive Substances (TBARS) assay was carried out for determining the lipid peroxidation in the lungsof rats exposed to hypoxia. The method involved heating-up of the assay mixture comprising of tissue homogenates, TBA, TCA and HCL in a boiling water bath at 80°C for 1 h. After 1 h, the assay mixture was allowed to cool down and centrifuged at 2000 rpm for 10 min. at 4°C. Later the absorbance of the supernatant obtained was measured spectrophotometrically (Synergy H1, Biotek, Germany) at 532 nm [32].

#### 2.7.5. Reduced glutathione (GSH) estimation

Quantification of GSH in the lungs of rats was carried out using5,5′-Dithiobis(2-nitrobenzoic acid) (DTNB) method reported by Tietze (1969) [33]and later modified by Adams et.al., (1983) [34]. The lung homogenate was precipitated with precipitating reagent, mixed thoroughly and incubated for 5 min. at room temperature. Later the reaction mixture was centrifuged at 1200 g for 20 min. at 4°C. The supernatant obtained was then mixed with phosphate buffer and DTNB reagent. Finally, the color developed was measured at 412 nm (OD) spectrophotometrically (Synergy H1, Biotek, Germany).

#### 2.7.6. Determination of pulmonary edema (vascular permeability and edema index)

The vascular permeability assay was performed with few modifications in the method as described by Schoch et.al. (2002) [35], using sodium fluorescein dye extravasation as a fluorescent marker for measuring transvascular leakage. Animals exposed to hypoxia (6h) were drawn out of the chamber half-an-hour before the exposure, administered intravenously with 1.5mg/Kg BW of sodium fluorescein dissolved in saline and were re-exposed to chamber for remaining 30min. of hypoxia exposure. After the completion of hypoxia exposure of 6h, animals were anesthetized and perfused with cold 1X PBS through left ventricles to clear out excess dye from the vascular bed. The lungs were then excised *en bloc*. One part of lung was placed in 3% formamide at room temperature for 18h. After 18h, the tissue was centrifuged at 3000 rpm for 10min. and the supernatant obtained was used for measuring the fluorescence at 530nm spectrophotometrically (Synergy H1, Biotek, Germany). Whereas, the other part of lung was weighed to record the wet weight of the lung and later kept in an oven for 72h at 80°C.Later the dry tissues were collected and weighed again to determine the dry weight. The results were presented as relative fluorescence units per gram (rfu/g) dry weight.

To estimate and compare the lung water content (edema index) in the lungs of both hypoxia exposed and unexposed animals, the wet weight of the tissues was measured immediately after removal. The lungs were washed thoroughly with cold 1X PBS and kept for 72h, at 80°C in hot air oven. After 72h the tissue was taken out from the oven, reweighed to register the dry weight of the lung and then edema index was expressed as wet to dry weight (W/D) ratio of the lungs [36].

#### 2.7.7. Glutathione peroxidise (GPx) estimation

The activity of the GPx in the lung homogenate of rats was measured by using commercial GPx diagnostic kit (Randox, UK), following the procedure mentioned in the manufacturer’s guidelines.

#### 2.7.8. Superoxide dismutase (SOD) estimation

SOD activity in the lungs of rats was estimated kinetically by using commercially available SOD diagnostic kit (Randox, UK), as per manufacturer’s instruction.

### 2.8. Protein expression studies

Nuclear extracts from lung homogenate of rats were obtained using nuclear/cytoplasmic fractionation kit (Biovision, CA., USA) according to manufacturer’s instructions. The protein content was quantified by Lowery’s method (1951) [37]. Further, we carried out western blotting analysis, to ascertain the effect of quercetin on elevated levels of NFĸB, Hif-1α and their related genes in lungs of rats exposed to hypoxia. The proteins in the samples were separated using 10% SDS-PAGE (IKKα/β, NFĸB, Nrf-2,His-H3,TNF- α, VEGF and β-actin) and 8% SDS-PAGE (Hif-1α, ICAM-1, VCAM-1, P-selectin and β-actin). The separated proteins were then electro-blotted onto the nitrocellulose membranes (0.45μm thickness) and blocked with 5% bovine serum albumin (BSA) dissolved in 1X PBS (pH ~7.4) overnight. Membranes were then washed and probed with primary antibodies (Santa cruz biotechnology, 1:5000 dilutions) and incubated for 2h at RT. Further, followed by the 4–5 washings using PBST the membranes were probed with HRP-conjugated, enzyme-linked secondary antibodies (Santa cruz, 1:15000 dilution) and incubated at room RT for 1h. After thorough washings (6–7 times) with PBST, the membranes were developed using chemiluminiscent peroxidase substrate (Luminata forte, Millipore, U.S.A.) and bands were visualized in Chemidoc (UVP, Cambridge, U.K.). The optical densityof bands were further quantified using lab works software (UVP-Bio-imaging systems, CA).

### 2.9. Broncho-alveolar lavage fluid extraction

BALF retrieval was carried out in all the animals under normoxia and hypoxia exposed groups. After 6h of hypoxia exposure,theanimals were anesthetized and an incision was made on the thorax region to expose trachea. The lungs were lavaged twice with 1ml saline (0.9% NaCl) using 18 guage micro-cannula through the lumen of exposed trachea. The fluid was retrieved and then centrifuged at 3000×*g* for 5 min. at 4°C. The supernatant obtained, was taken into different wells of 96-well plate (Tarsons Pvt. Ltd., India), in triplicate for the estimation of total protein content present in the BAL fluid of the rats using Lowery method [37,38].

#### 2.9.1. Estimation of pro-inflammatory cytokines (TNF-α and INF-γ) and anti-inflammatory cytokines (TGF-β and IL-4) from BALF

The expressions of the pro-inflammatory and anti-inflammatory cytokines in BAL fluid of the animals exposed to normoxia and hypoxia groups were quantified by using rat TNF-α and INF-γ ELISA set (B.D. Biosciences, U.S.), whereas the TGF-β and IL-4 estimation was carried out using standard rat TGF-β ELISA kit (R&D systems, Canada, U.S.) and rat IL-4 ELISA kit (B.D. Biosciences, U.S.). The assays were performed according to manufacturer’s instructions.

#### 2.9.2. Quantification of Lactate dehydrogenase (LDH) and Albumin content in BAL fluid

The LDH levels were measured as biomarker of cytotoxicity. Concentrations of LDH in the BALF of rats were estimated using an enzyme based cytotoxicity detection kit (Biovision Inc., U.S.) according to manufacturer’s instructions.

Albumin content in the BALF serves as an indicator of microvascular permeability, was determined by using commercially available ELISA kit (ICL, U.S.A.) according to manufacturer’s instructions.

### 2.10. Histopathological examination

Histological examination was done by staining the lung sections from different groups using hematoxylin and eosin (H & E) stain. For histopathological examination, animals were anesthetized, the lungs were quickly excised and fixed in 10% formalin for 24h. The fixed tissues were later truncated into fine sections (5μm thickness) and stained with H & E stain. Finally, the micrographic images were captured using an Olympus BX 50 microscope (Olympus, Japan).

### 2.11. Statistical analysis

Statistical analysis was performed using SPSS for Windows (15.0) software (SPSS Inc., Chicago, IL). Comparisons between experimental groups and quercetin pretreated groups were made by using one-way ANOVA with Student-Newman-Keuls test for multiple comparisons between groups. Whereas, comparisons between normoxia-exposed (0 hrs), hypoxia-exposed (6 h) animals and hypoxia + quercetin treated groups were made using Student’s t test. Differences were considered statistically significant for p<0.001. Results were expressed as mean ± SD.

## 3. Results

### 3.1. Altercations in biochemical parameters

#### 3.1.1. Reactive oxygen species (ROS)

**Fig 1:**
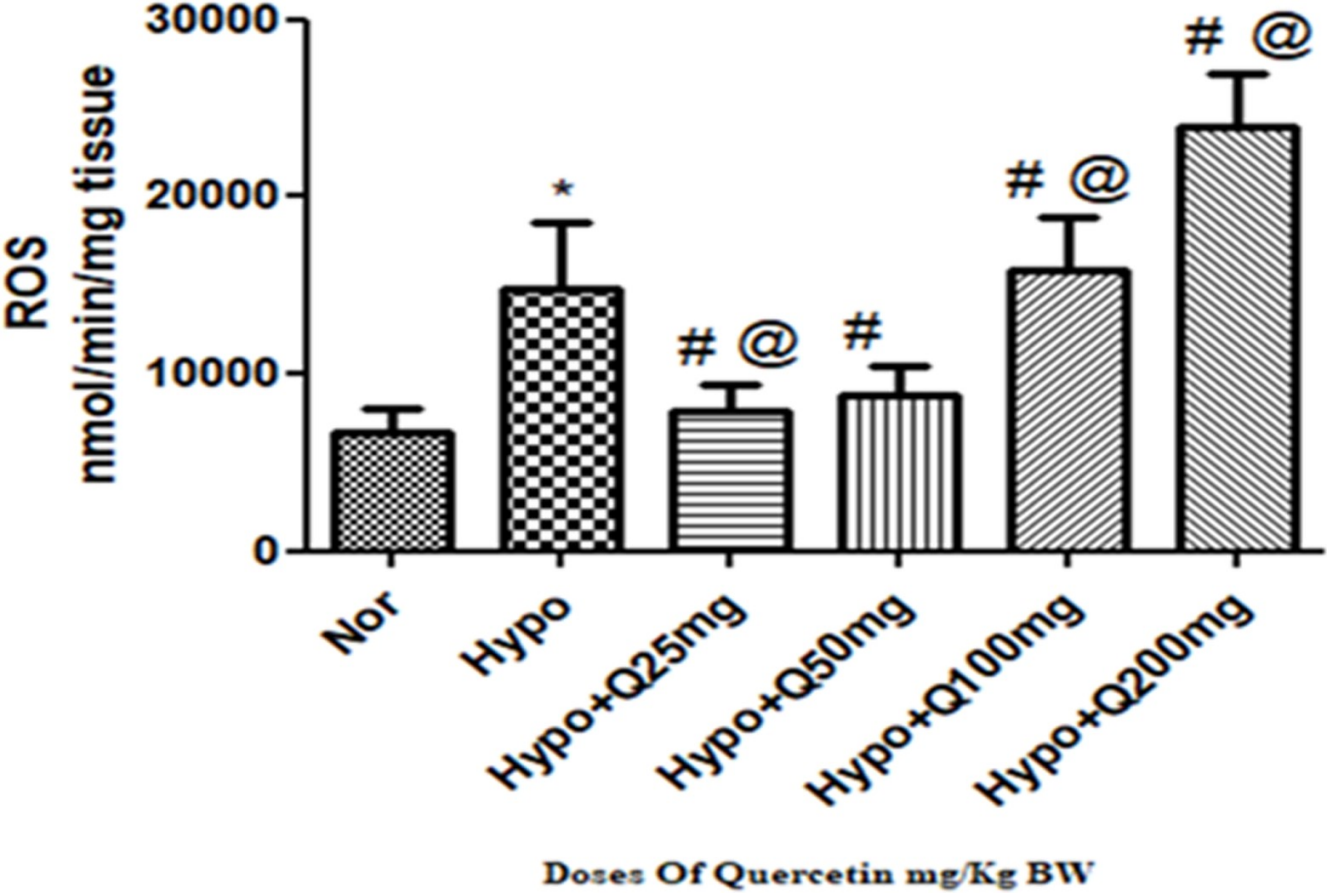
Effect of different doses of quercetin (25mg, 50mg, 100mg, 200mg/Kg BW) on Reactive oxygen species (ROS) generation in lungs of rats exposed to hypoxia at 7,620m for 6h. Values are mean±SD (n = 6). *P<0.001 normoxia vs hypoxia group, #P<0.05 groups compared with hypoxia and @P<0.05 groups compared with hypoxia+quercetin (50 mg/kg BW). Nor-Normoxia, Hypo-Hypoxia and Q 25, 50, 100 and 200mg- Quercetin @ 25, 50, 100 and 200 mg/kg BW.

A significant increase (p<0.001) (2-fold ↑) observed in the levels of ROS generation in lungs of rats exposed to hypoxia as compared to normoxia control (0h). Whereas, the supplementation of quercetin @ 25mg and 50 mg/kg BW has significantly (p<0.001) reduced the levels of ROS (2-folds ↓) compared to control (6h) respectively. However, with further increase in the dosage of quercetin above 50 mg/kg BW i.e., in 100mg/kg BW and 200mg/Kg BW, a significant elevation in the generation of ROS was observed compared to hypoxia control (6h) (Fig 1).

#### 3.1.2. Changes in lung lipid peroxidation(MDA) levels

**Fig 2:**
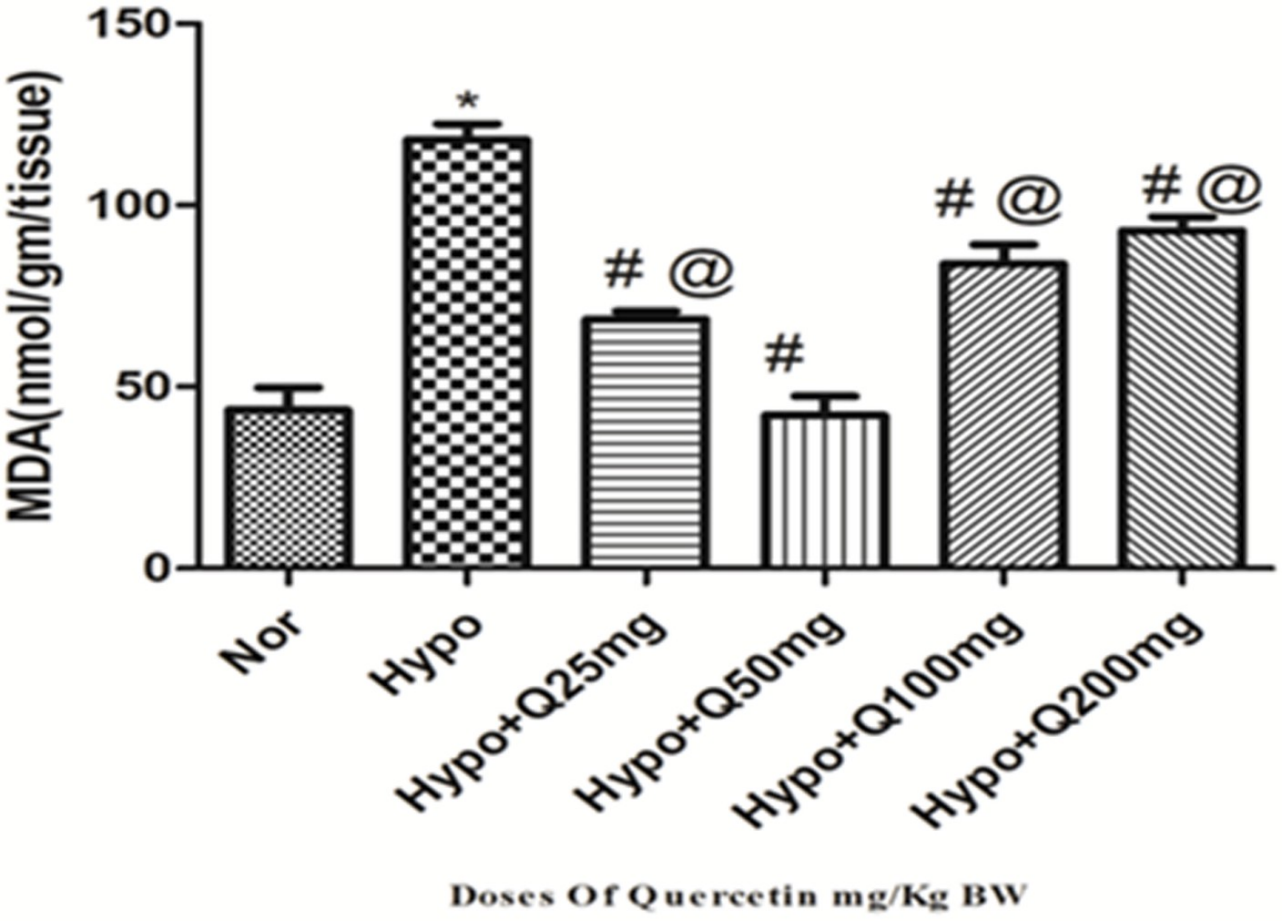
Effect of different doses of quercetin (25mg, 50mg, 100mg, 200mg/Kg BW) on lipid per-oxidation (MDA) in lungs of rats exposed to hypoxia at 7,620m for 6h. Values are mean±SD (n = 6). *P<0.001 normoxia vs hypoxia group, #P<0.05 groups compared with hypoxia and @P<0.05 groups compared with hypoxia+quercetin (50 mg/kg BW). Nor-Normoxia, Hypo-Hypoxia and Q 25, 50, 100 and 200mg- Quercetin @ 25, 50, 100 and 200 mg/kg BW.

There was a significant up regulation (p<0.001) in the levels of MDA (2.5-folds ↑) observed in the lung of rats exposed to hypoxia (6h) compared to normoxia control (0h). Whereas, quercetin (50 mg/Kg BW) supplementation 1h prior to hypoxia exposure has appreciably reduced (p<0.001) (2.5-folds ↓) the MDA levels compared to hypoxia control (6h). Whereas, other doses of quercetin @25mg, 100mg and 200mg/kg BW (2-folds ↓, 1.2-folds ↓ and 1-folds ↓respectively) also showed reduction in MDA levels compared to hypoxia control (6h), however among all these doses, 50 mg/kg BW quercetin exhibited more significant and better reduction in MDA levels compared to hypoxia control but more or less similar to normoxia control (0h) (Fig 2).

#### 3.1.3. Modulation in GSH levels

**Fig 3:**
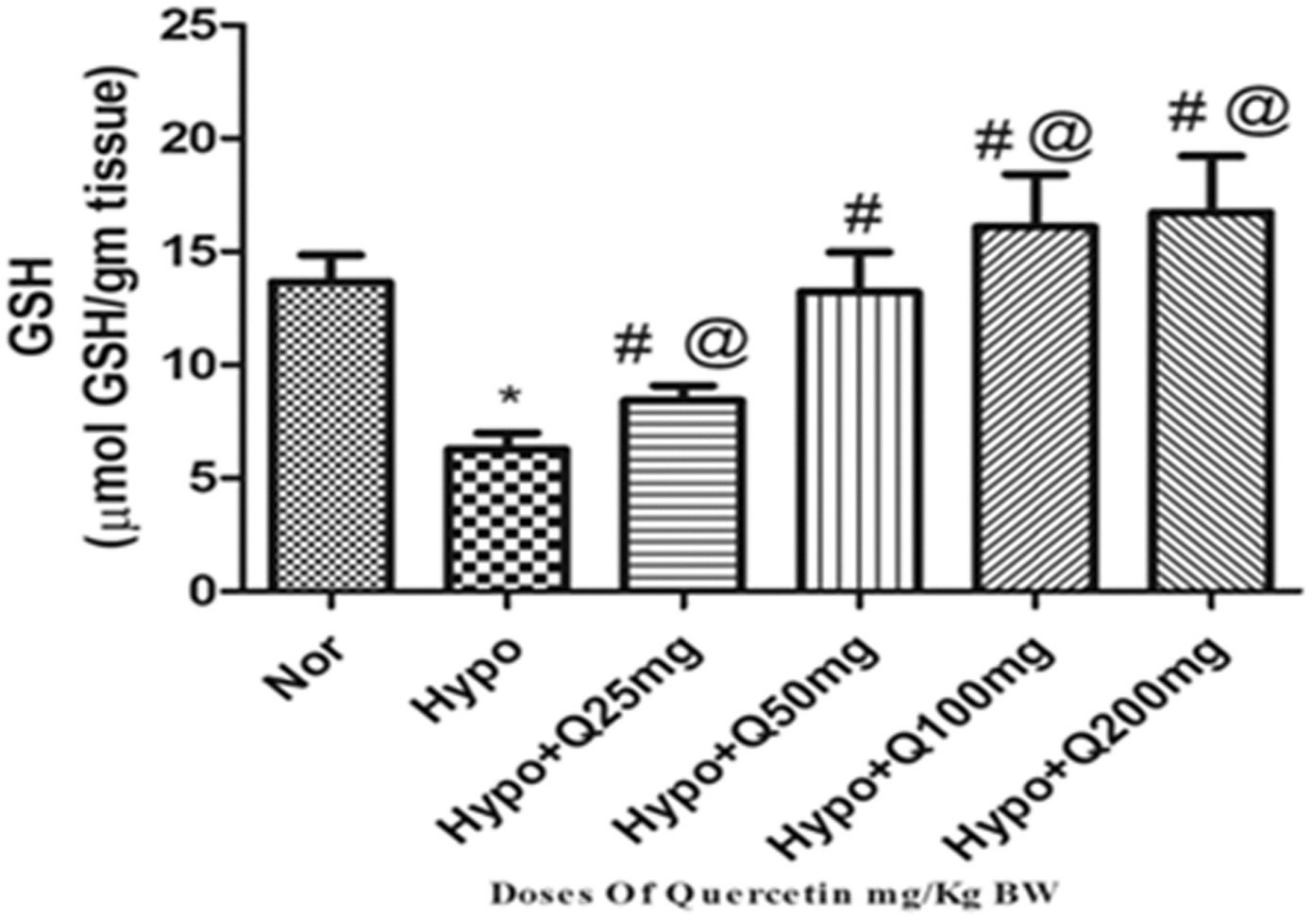
Effect of different doses of quercetin (25mg, 50mg, 100mg, 200mg/Kg BW) on reduced glutathione (GSH) levels in lungs of rats exposed to hypoxia at 7,620m for 6h. Values are mean±SD (n = 6). *P<0.001 normoxia vs hypoxia group, #P<0.05 groups compared with hypoxia and @P<0.05 groups compared with hypoxia+quercetin (50 mg/kg BW). Nor-Normoxia, Hypo-Hypoxia and Q 25, 50, 100 and 200mg- Quercetin @ 25, 50, 100 and 200 mg/kg BW.

Animals exposed to hypoxia demonstrated a significant reduction (p<0.001) in GSH levels (2.5-folds ↓) compared to normoxiacontrol (0h). The supplementation of 25mg/Kg BW dose of quercetin has demonstrated a non-significant elevation in GSH levels compared to hypoxia control (6h). Whereas, prophylactic administration of quercetin (50 mg/Kg BW) has significantly elevated (p<0.001) the GSH levels (2.5-folds ↑) compared to hypoxia control (6h). However, quercetin with 100mg and 200mg/Kg BW exhibited further significant increase (p<0.001) (3-folds ↑) in GSH levels as compared to hypoxia control (6h) (Fig 3).

#### 3.1.4. Determination of pulmonary edema (lung water content and transvascular leakage)

**Fig 4:**
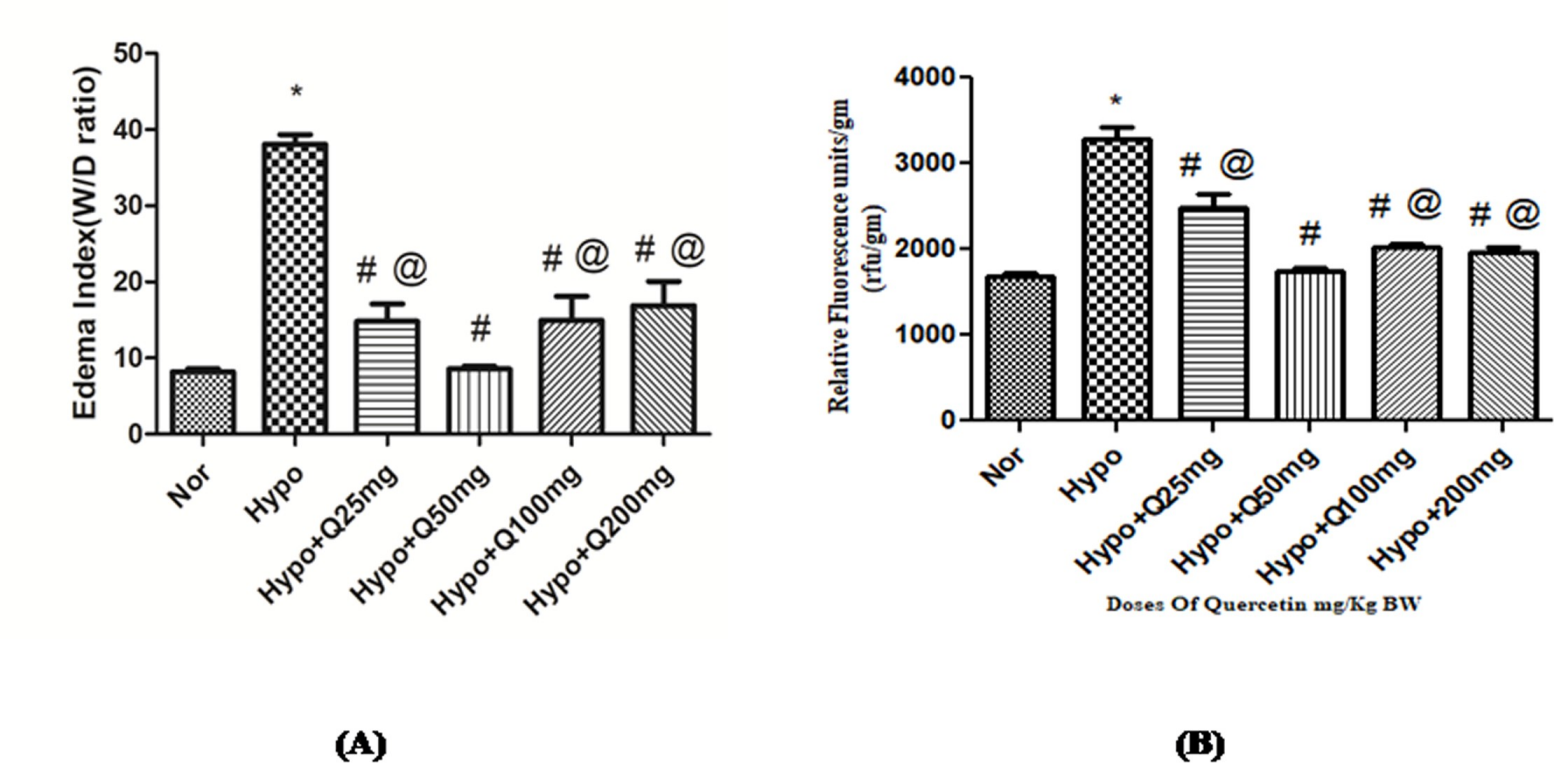
Standardization of quercetin dose (25mg, 50mg, 100mg, 200mg/Kg BW) in hypoxia (7,620m for 6h) exposed animals (A) Edema index (B) Transvascular leakage. Values are mean±SD (n = 6). *P<0.001 normoxia vs hypoxia group, #P<0.05 groups compared with hypoxia and @P<0.05 groups compared with hypoxia+quercetin. Nor-Normoxia, Hypo-Hypoxia and Q 25, 50, 100 and 200mg/Kg BW- Quercetin @ 25, 50, 100 and 200 mg/kg BW.

Hypoxia exposure of 6h has delineated the significant increase (p<0.001) in lung water content (38.05±2.8W/D ratio) over normoxia control (8.5±0.5 W/D ratio), which was quantified by estimating the wet weight by dry weight (W/D) ratio of the lungs of rats. Whereas, the animals supplemented with 25mg, 100mg and 200mg/Kg BW of quercetin resulted into significant reduction (p<0.001) (14±5, 14.93±7.1 and 16.9±7 W/D ratio respectively) in the lung water content compared to hypoxia control (6h). However, the prophylactic administration of quercetin with 50 mg/kg BW to the animals exposed to hypoxia showed the significant decrease (p<0.001) in the edema index (8.2±1 W/D ratio) observed over hypoxia control (6h) (Fig 4(A)). However, these results were further confirmed by vascular leakage study.

Animals exposed to hypoxia showed a significant increase (p<0.001) in transvascular leakage (3290.17±106rfu/gm tissue) over normoxia control (0h) (1689.5±60.63rfu/gm tissue). However, quercetin supplementation (50 mg/kg BW) resulted into significant attenuation (p<0.001) in the transvascular leakage (1752.33±53 rfu/gm tissue) compared to hypoxia exposed rats. Whereas, the animals fed with 25mg, 100mg and 200mg/Kg BW of quercetin exhibited the relative fluorescence of 2824±98.8, 2032.78±82 and 1982.26±91rfu/gm tissue (Fig 4(B)) respectively. Quercetin (50 mg/kg BW) supplementation under the hypoxic condition resulted into the reduced ROS generation, attenuated MDA levels, enhanced GSH and GPx levels, with reduced vascular leakage and lung water content. Therefore, considering all the biochemical parameters, the optimum dose of quercetin was found to be 50 mg/kg BW. Based on these findings, the rest of the study was carried out using 50 mg/kg BW of quercetin as an optimum dose.

#### 3.1.5. Effect of quercetin on GPx activity in the lungs of rats exposed to hypoxia

**Fig 5:**
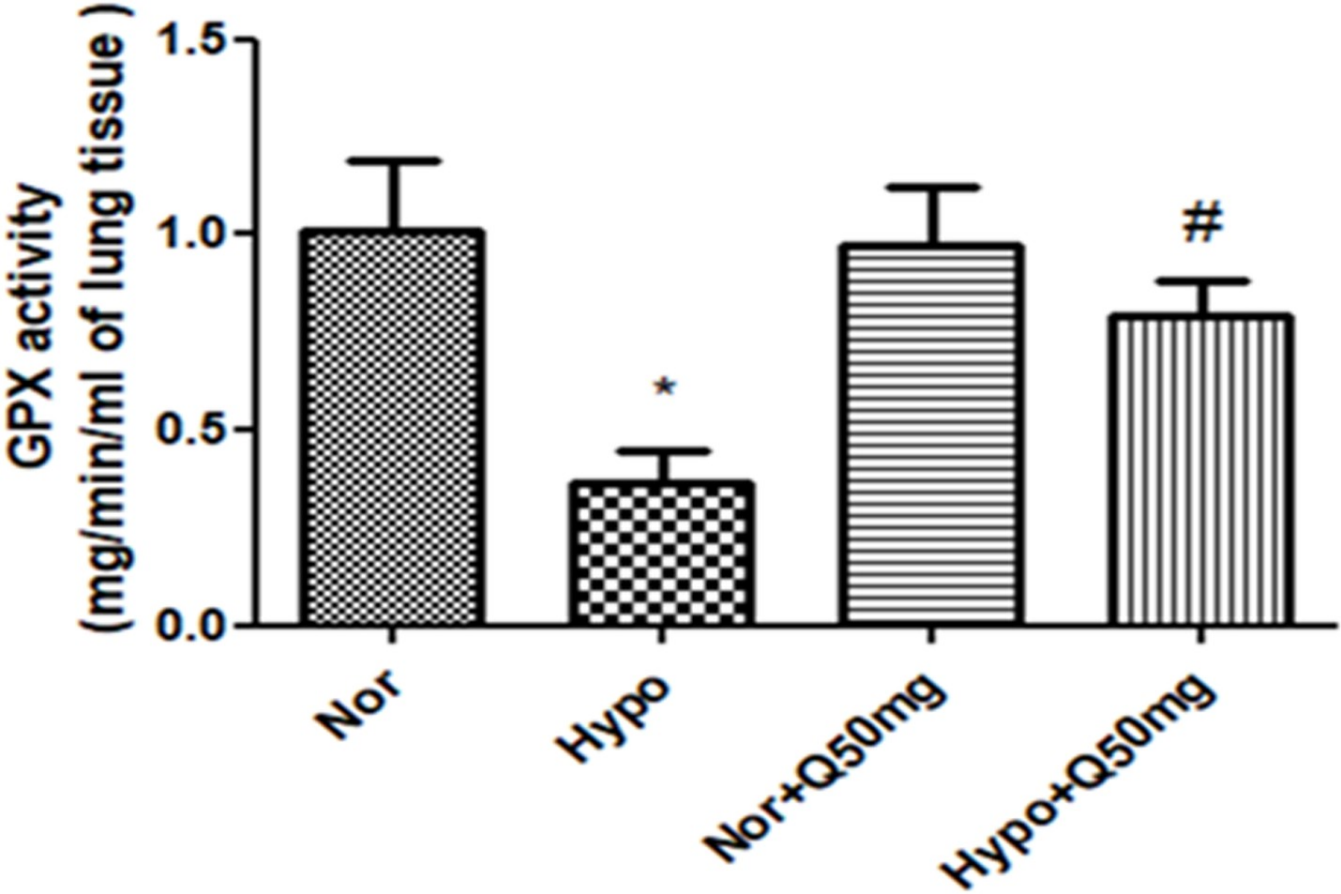
Effect of quercetin (50 mg/kg BW) prophylaxis on the activity of glutathione peroxidase (GPx) presentin the lungs of rats exposed hypoxia at 7,620 m for 6 h. Values are mean±SD (n = 6). *P<0.001 normoxia vs hypoxia, #P<0.05 hypoxia vs hypoxia+quercetin (50 mg/kg BW). Nor-Normoxia, Hypo-Hypoxia and Q50mg- Quercetin 50mg/kg BW.

The activity of glutathione peroxidase was found to be significantly down regulated (p<0.001) (2.5-folds ↓) in lungs of the animals exposed to hypoxia over normoxia control (0h). However, the animals receiving quercetin (50mg/ Kg BW) 1h prior to hypoxia exposure (6h) exhibited a significant increase (p<0.001) in the GPx activity (2- folds ↑) compared to hypoxia control (6h). Whereas, the animals fed with quercetin (50 mg/kg BW) under normal condition did not show any significant alteration in GPx activity compared to normoxia (Fig 5).

#### 3.1.6. Effect of quercetin on lung SOD activity under hypoxia

**Fig 6:**
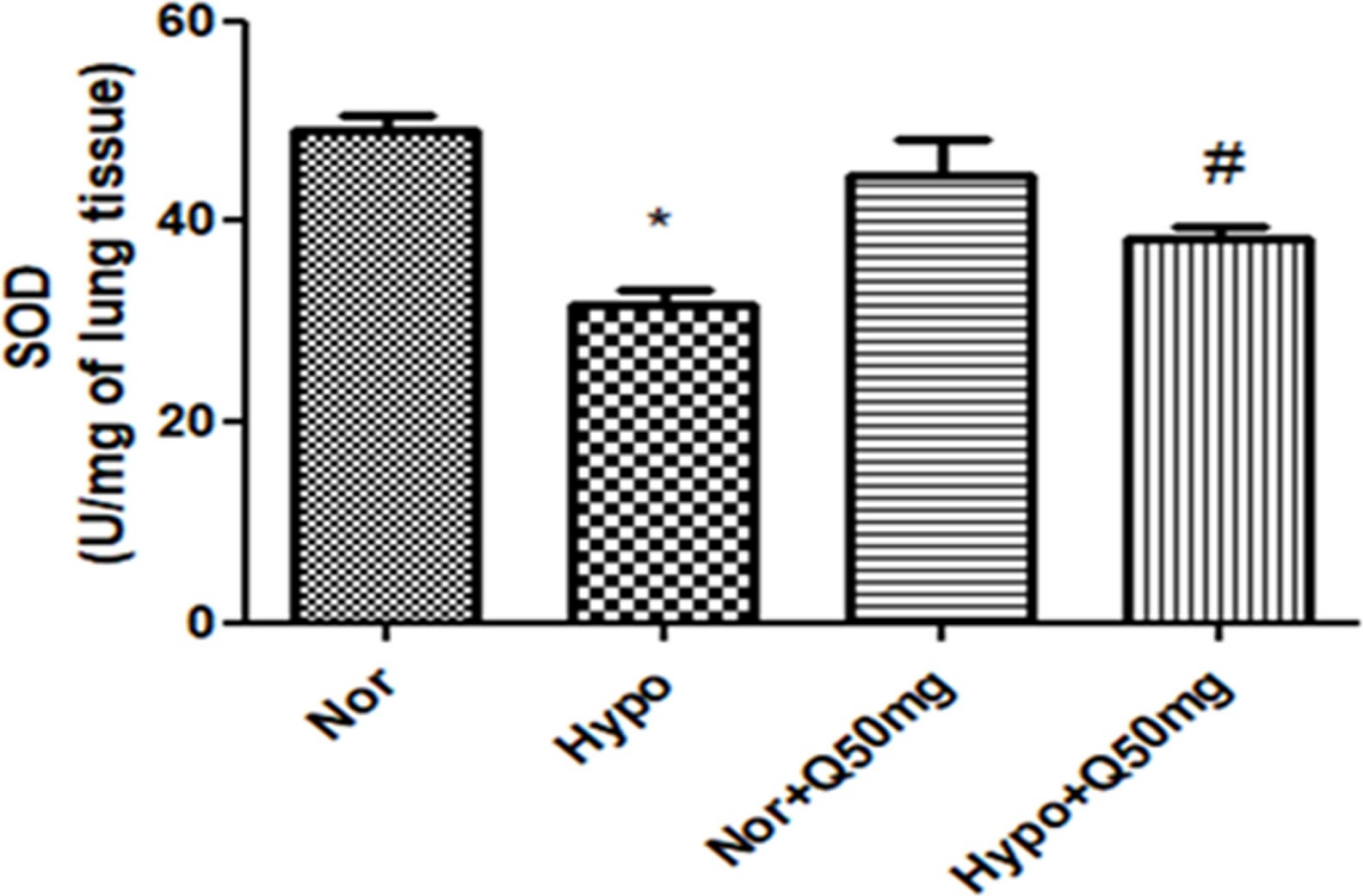
Effect of quercetin (50 mg/kg BW) prophylaxis on the activity of SOD in the lungs of rats exposed to hypoxia at 7,620 m for 6 h. Values are mean±SD (n = 6). *P<0.001 normoxia vs hypoxia, #P<0.05 hypoxia vs hypoxia+quercetin (50 mg/kg BW). Nor-Normoxia, Hypo-Hypoxia and Q50mg- Quercetin 50mg/kg BW.

Rats exposed to hypoxia demonstrated the significant reduction (p<0.001) in SOD activity (1.5-folds ↓) compared to normoxia control (0 h). Whereas, the supplementation of quercetin (50 mg/kg BW) showed significant up regulation (p<0.001) in SOD activity (1.4-folds ↑) over hypoxia control (6 h). However, no significant change in the activity was observed in lungs of rats supplemented with quercetin (50 mg/kg BW) under normoxic condition (Fig 6).

### 3.2. Effect of quercetin on protein expressions of transcriptional factors NFĸB, HIF-1α, Nrf-2& their regulatory genes and associated cell-adhesion molecules (ICAM-1, VCAM-1 and P-selectin) in lungs of rats under hypoxia

**Fig 7:**
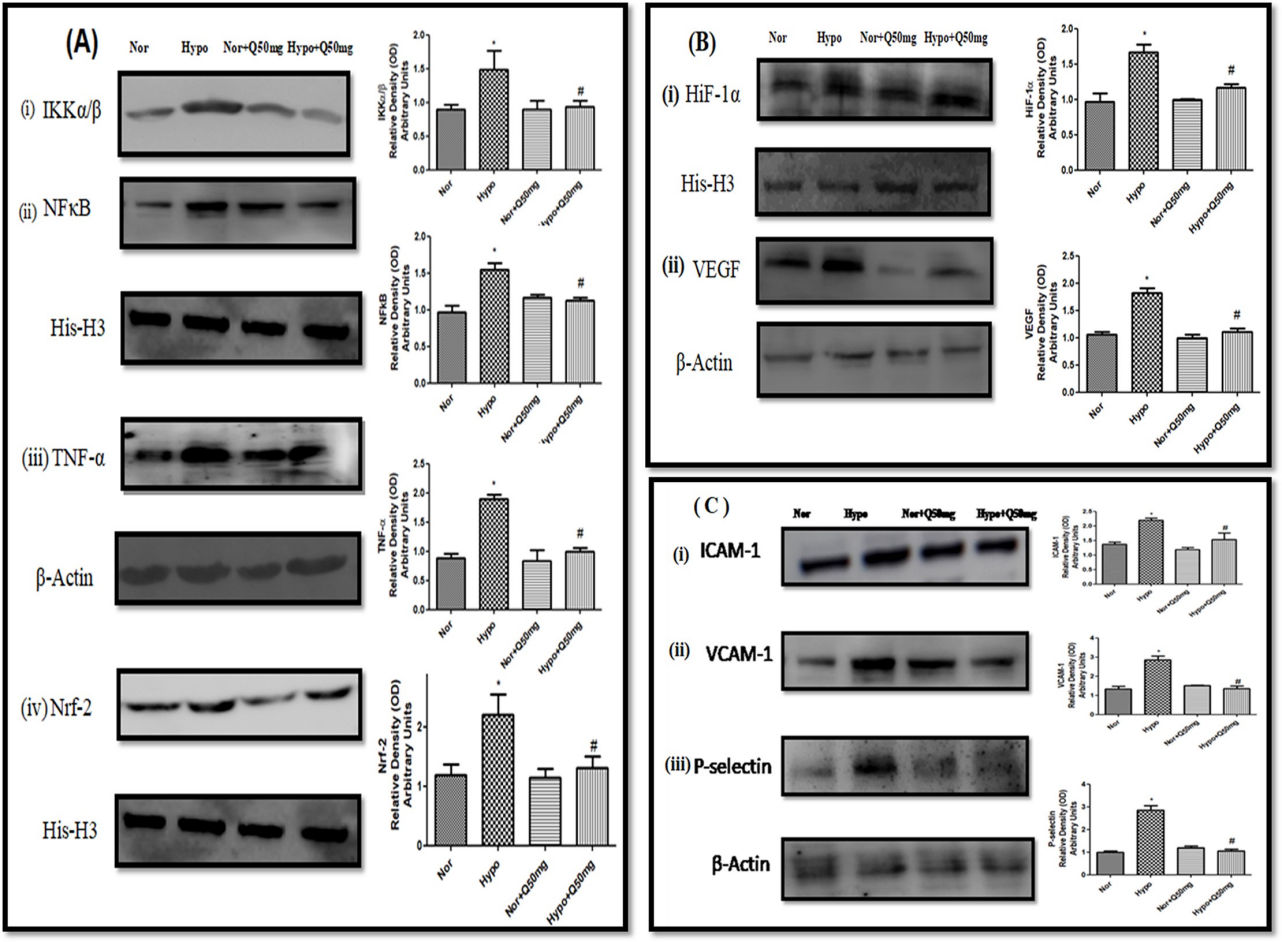
Efficacy of quercetin (50 mg/kg BW) supplementation on the expressions of:(A) (i) IKK-α/β, (ii) NFĸB (iii) TNF-α and (iv) Nrf-2, (B) (i) Hif-1α and (ii) VEGF expressions and (C) (i) ICAM-1 (ii) VCAM-1 and (iii) P-selectin in lungs of the rats exposed to hypoxia at 7,620 m for 6 h. Values are mean±SD (n = 6). *P<0.001 normoxia vs hypoxia, #P<0.05 hypoxia vs hypoxia+quercetin (50 mg/kg BW). Nor-Normoxia, Hypo-Hypoxia and Q50mg- Quercetin 50mg/kg BW.

The levels of IKKα/β, NFĸB and Nrf-2 in the lung homogenates of hypoxia exposed animals were found to be up regulated significantly (p<0.001) (3-folds ↑, 2-folds ↑ and 2-folds η,respectively) compared to normoxia control (normoxia). Whereas, supplementation of quercetin (50 mg/kg BW) resulted into the down regulation in the levels of IKKα/β (2.5 folds ↓),NFĸB (1.6-folds ↓) and Nrf-2 (1.75-folds ι) significantly (p<0.001) compared to hypoxia control (6h) (Fig 7(A) (i),(ii) and (iv) respectively). In addition to this, protein expression studies of TNF-α also resembled the similar pattern of protein expressions as that of NFĸB (Fig 7(A) (iii)).The densitometric analysis of IKKα/β, NFĸB, TNF-α and Nrf-2 have been reported alongside of each blot.

However, the levels of Hif-1α and its downstream gene VEGF in the lungs of the rats exposed to hypoxia demonstrated a significant increase (p<0.001) (1.7-folds ↑ and 1.6-folds ↑ respectively) compared to normoxia control (0h). Whereas, the quercetin (50 mg/kg BW) supplementation resulted into stabilized Hif-1α expression and significantly reduced the VEGF (P<0.001) (1.5-folds ↓) expressions compared to hypoxia control (6h) (Fig 7(B) (i) and (ii) respectively). The densitometric analysis of Hif-1α and VEGF has been reported alongside of each blot.

Moreover, the hypoxia exposure has also resulted into the significant increase (p<0.001) in the expression of cell adhesion molecules (ICAM-1, VCAM-1 and P-selectin) (1.2-folds ↑, 2-folds ↑ and 3-folds η respectively) compared to normoxia control (0h). Whereas, the prophylactic administration of quercetin demonstrated a significant reduction (p<0.001) in their levels (1.5-folds ↓, 2-folds ↓ and 3-folds ↓respectively) compared to hypoxia control (6h)(Fig 7(C)(i), (ii) and (iii) respectively). The densitometric analyses of ICAM-1, VCAM-1 and P-selectin have been reported alongside of each blot.

However, the animals fed with same dose of quercetin under normal condition exhibited unmodified expressions of IKKα/β, NFĸB, Nrf-2, and TNF-α, Hif-1α,and VEGF, ICAM-1, VCAM-1 and P-selectin.

### 3.3. Modulation in the expressions of pro-inflammatory and anti-inflammatory cytokines by quercetin prophylaxis

**Fig 8:**
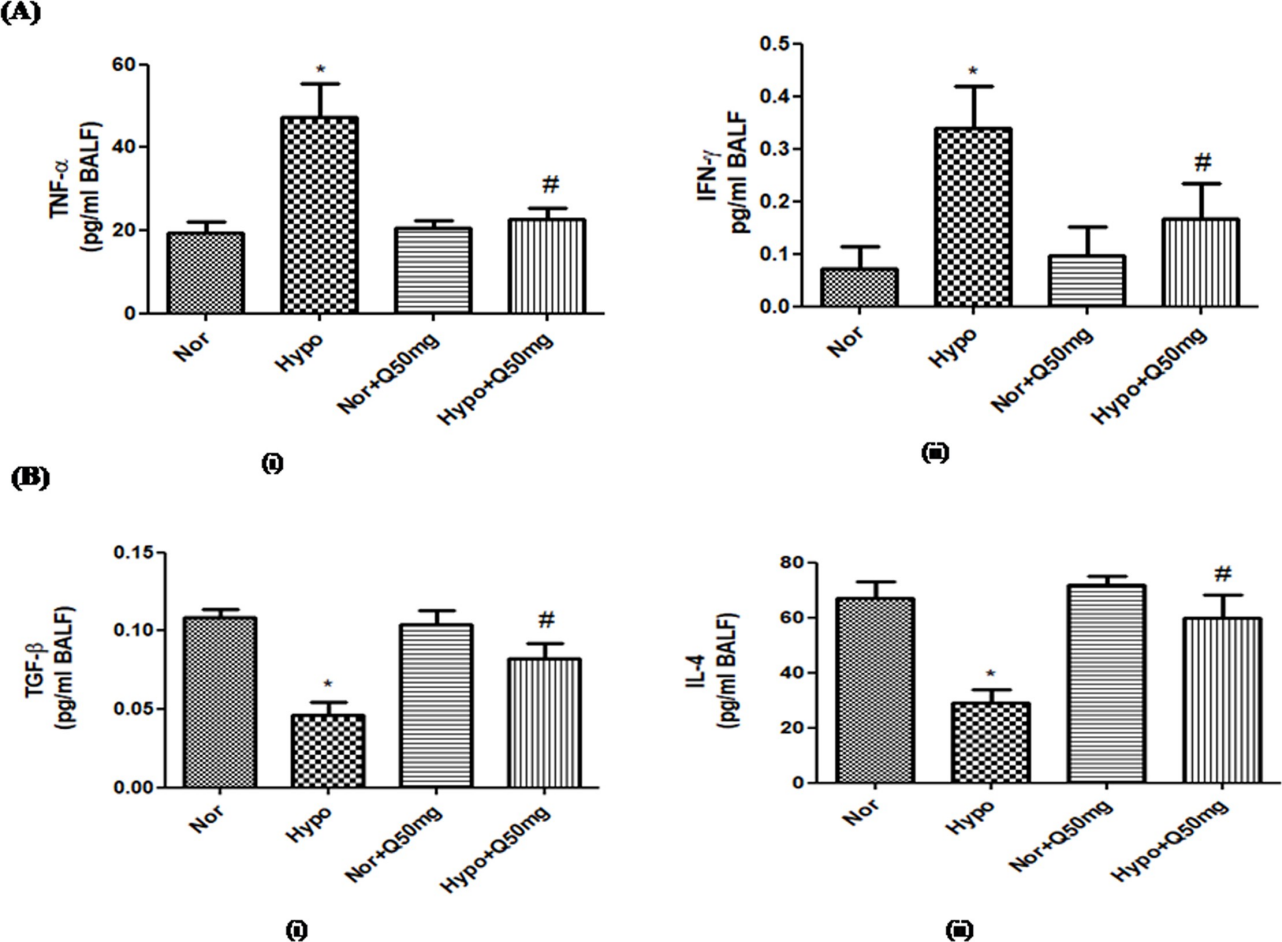
Effect of prophylactic administration of quercetin (50 mg/kg BW) on: (A) pro-inflammatory ((i) TNF-α and (ii) INF-γ) and (B) anti-inflammatory cytokines ((i) TGF-β and (ii) IL-4) expressions in lung of rats exposed to hypoxia at 7,620m for 6h. Values are mean±SD (n = 6). *P<0.001 normoxia vs hypoxia, #P<0.05 hypoxia vs hypoxia+quercetin (50 mg/kg BW). Nor-Normoxia, Hypo-Hypoxia and Q50mg- Quercetin 50mg/kg BW.

The hypoxia exposure has resulted into significant up regulation (p<0.001) in the expression of pro-inflammatory cytokines (TNF-α and INF-γ) (2.5-folds ↑ and 2-folds ↑respectively)compared to normoxia control (0h). Whereas, the expressions of anti-inflammatory cytokines (TGF-β and IL-4) in BAL fluid of hypoxia exposed animal was found to be significantly down regulated (P<0.001) compared to normoxiacontrol (0h) (2.5-folds↓ and 2-folds↓respectively). However, the quercetin preconditioning demonstrated the significant attenuation (p<0.001) in the expressions of TNF-α and INF-γ (2-folds↓ and 1.6-folds ↓ respectively) compared to hypoxia control (6h) (Fig 8(A)(i) and (ii) respectively). While, the expressions of TGF-β and IL-4 were found to be elevated (1.5-folds↑ and 1.5-folds ↑ respectively) significantly (p<0.001) in the quercetin pre-treated groups compared to hypoxia control (6h). However, unmodified expressions of pro-and anti-inflammatory cytokines were observed in the BAL fluid of the animals recieving quercetin (50 mg/kg BW) under normoxia(Fig 8(B) (i) and (ii) respectively).

#### 3.3.1. Effect of quercetin on LDH and Albumin content in the BAL fluid

**Fig 9:**
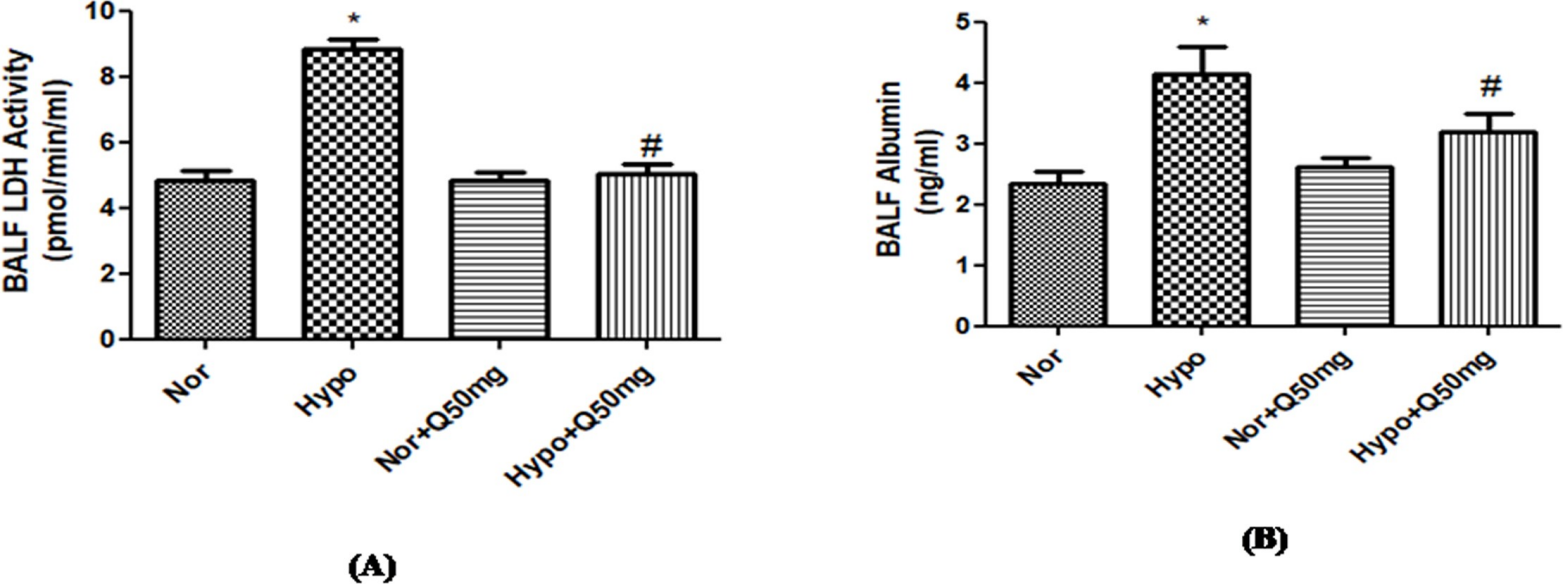
Effect of quercetin (50 mg/kg BW) prophylaxis on the (A) LDH and (B) Albumin extravasation into the lungs of rats exposed at 7,620m for 6h. Values are mean±SD (n = 6). *P<0.001 normoxia vs hypoxia, #P<0.05 hypoxia vs hypoxia+quercetin (50 mg/kg BW). Nor-Normoxia, Hypo-Hypoxia and Q50mg- Quercetin 50mg/kg BW.

BALF of the hypoxia exposed animals showed a significant increase (p<0.001) in both LDH and albumin content (2-folds ↑ and 2-folds ↑ respectively) compared to the animals under normoxia condition. Whereas, the pre-conditioning with quercetin (50 mg/kg BW) 1h prior to hypoxia exposure resulted into significant down regulation (p<0.001) in the LDH (2-folds↓) and albumin (2-folds↓) contents in BAL fluid of the rats exposed to hypoxia compared to the hypoxic control (6h). However, the BALF content of the animals fed with quercetin (50 mg/kg BW) under normoxic condition showed no changes in both LDH and albumin contents in the lungs compared to control (0h) (Fig 9(A) and 9(B)).

### 3.4. Histopathological changes of lung tissue under Quercetin prophylaxis

**Fig 10:**
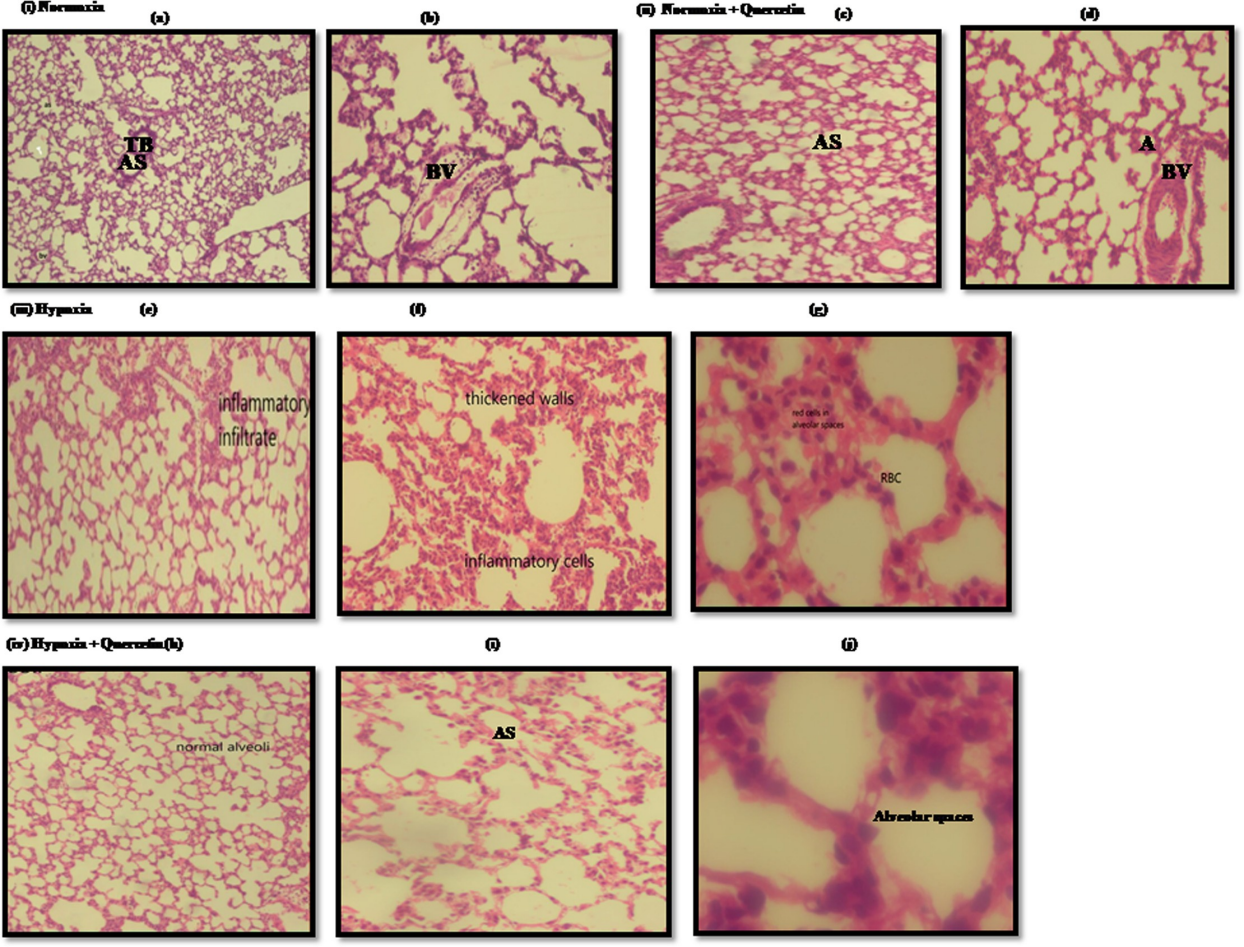
Histopathological images representing the collapsed alveoli, infiltration of inflammatory cells and RBCs, ruptured blood vessels and thin septae in lung of rats exposed to hypoxia at 7,620m for 6h. (i) (a) and (b) Low power photomicrograph (10X) of lung section from normoxia control group (0h) representing normal lung parenchyma, clear AS and normal TB(ii) (c) Low power photomicrography of lung parenchyma of normoxic animal fed with Quercetin (50mg/kg BW), showed normal lung configuration with medium sized alveoli and normal blood vessels (d) High power photomicrography (40X) from the same section, exhibited normal alveolar spaces surrounded by medium septae (iii) (e) Low power photomicrograph of (10X) of lung section of hypoxic animal (hypoxia control (6h)) demonstrated the infiltration of infiltatory cells (f) High power photomicrographs (40X) of another lung section of hypoxic animal exhibiting the inflammation and thickened walls (g) High power photomicrograph (100X) of another lung section demonstrated the appearance of RBCs in alveolar spaces (iv) (h) Low power photomicrograph (10X) of lung parenchyma of hypoxic animal supplemented with quercetin (50mg/kg BW) showed normal alveolar structure (i) High power photomicrograph (40X) from the same section showing clear alveolar spaces and blood vessels (j) High power photomicrograph (100X) from the same section of hypoxia +Quercetin animal lung, manifested clear alveolar spaces devoid of any appearance of inflammatory cells and RBCs.where A- alveoli, AS- alveolar spaces, TB- terminal bronchiole, ICs- Inflammatory cells.

The histopathological examination of normoxic group manifested the normal configuration of the lung parenchyma (Fig 10(i)(a) and (b)).The lung sections of the animals under normoxia+quercetin group exhibited the pattern similar to normoxic lung (Fig 10(ii) (c) and (d)).Whereas, the hypoxic lung sections have shown the presence of collapsed alveoli, infiltration of inflammatory cells, thickened inter-alveolar walls and appearance of red blood cells (RBCs) in alveolar spaces (Fig 10 (iii) (e), (f) and (g)). However, the lung sections of the animals fed with quercetin prior to hypoxia demonstrated normal alveoli, reduced infiltration of inflammatory cytokines and disappearance RBCs in alveolar spaces (Fig 10 (iv) (h), (i) and (j)).

## 4. Discussion

Quercetin has been reported to be a potent anti-inflammatory and anti-oxidant biomolecule with bioprotective and phytonutrient properties [39,40]. However, there is not much information available about the molecular mechanism of quercetin against non-cardiogenic form of pulmonary edema. Therefore, we undertook an investigation to find out the prophylactic efficacy of quercetin in combating the hypoxia induced pulmonary edema in rats exposed to high altitude.

Non-cardiogenic pulmonary edema occurs due to increased permeability of microvascular and alveolar compartments with accumulation of highly concentrated plasma proteins (specifically, albumin), as a consequence of hypoxic insult [41]. The major finding of this study was that the prophylactic administration of quercetin in ameliorating the transvascularleakge in the lungs of rats under hypoxia was attained by attenuation of hypoxia induced oxidative stress and inflammation. We proposed the following molecular mechanism in order to achieve the reduction in fluid influx into the lungs of rats by quercetin prophylaxis at high altitude: (1) Quercetin prophylaxis significantly attenuates the inflammation (NFĸB). This attenuation seems to occur at upstream of NFĸB at IKKα/β level (2) Attenuation of NFĸB further significantly down regulated the proinflammatory cytokines (TNF-α & IFN γ) followed by elevated proinflammatory cytokines (TGF- β& IL-4). (3). Preconditioning with quercetin, stabilized Hif-1 α followed by reduction in its angiogenic regulatory gene i.e., VEGF (4) Quercetin prophylaxis reduced the fluid build-up in to the lungs of rats exposed to hypoxia.

Literature has revealed that the excessive production of ROS in the hypoxic environment is one of the prominent factors responsible for oxidative damage [42]. Emergences of free radicals are implicated to damage the biological membranes which precede to compromise cellular integrity and function [43]. Earlier studies have also evidenced that, besides the exceeding pulmonary artery pressure, exaggerated free radical generation under hypoxic environment may also leads to oxidative injury of endothelium resulting into elevated pulmonary capillary permeability [44]. The oxidative damage in proximity with activated inflammatory cells is reported to cause modulations in Na^+^ reabsorption in lungs along with the alterations in certain signaling cascades such as- β2-AR pathways [45]. In contrast to this, we have also showed that hypoxia aggravates generation of free radicals, lipid peroxidation and reduction in anti-oxidants, which altogether gets normalized after quercetin supplementation prior to hypoxia exposure i.e., attenuation of ROS generation, reduction of lipid per-oxidation and increased synthesis of reduced glutathione (GSH). Another reason for the up regulation in GSH levels upon quercetin prophylaxis is the increased synthesis of Nrf-2 leading to the activation of nuclear factor (erythroid-derived-2)-like 2/antioxidant responsive element (Nrf-2/ARE) pathway. GSH, is a tripeptide (made up of glutamate, cysteine and glycine) synthesized by the sequential action of two rate limiting enzymes- γ- glutamyl cysteine ligase and glutathione synthetase [46]. GSH is mainly attributed with the non-enzymatic detoxification of ROS such as- hydroxyl radicals and superoxidesand also donates an electron to reduce the peroxides catalysed by glutathione peroxidases [47]. γ- glutamyl cysteine ligase is largely responsible for controlling the rate of GSH synthesis and the expression of γ- glutamyl cysteine ligase is reported to be regulated by Nrf-2, a redox-sensitive transcriptional factor [48,49]. The studies on Nrf-2 have revealed that under basal conditions, Nrf-2 remains associated with kelch-like-ECH-associated protein1 (Keap1), which promotes the proteosomal mediated ubiquitination of Nrf-2 [49]. Whereas, during oxidative stress conditions, Keap1 gets dissociated from Nrf-2 and enables to get translocated into the nucleus, where, Nrf-2 interacts with ARE and boosts up the synthesis of GSH [50]. In the present study quercetin was found to enact as an Nrf-2 activator in the hypoxic condition which further catalyzed the significant elevation in the levels of anti-oxidant such as- GSH.

Not only this, another finding obtained from the present study was that, by exceeding the dose above 50 mg/kg BW of quercetin i.e., 100mg and 200mg/Kg BW, an increment in the levels of ROS were observed in the lungs of rats exposed to hypoxia compared to hypoxia control (6h), indicating the pro-oxidant activity of quercetin at higher doses. However, in correlation with these results, the MDA levels were found to be reduced significantly in all the four doses tested compared to hypoxia control (6h). Earlier it was reported that, reaction of oxygen with unsaturated lipids or lipid per-oxidation, generates an extensive variety of oxidation products [51]. Among these products, lipid hydroperoxides are the main primary products, whereas, malondialdehyde (MDA), hexanal and 4-hydroxynonenal (4-HNE) are most widely studied secondary outcomes of lipid peroxidation [52]. MDA is mainly considered as an end-product generated from the enzymatic and/or non-enzymatic decomposition of larger PUFAs and arachidonic acid (AA) [53]. MDA is also known as a major metabolite of AA and a biomarker of oxidative stress [54]. Esterbauer et.al., (1985) reported that MDA is considered to be membrane-permeable and more stable than ROS [53]. Enzymatically, MDA can be generated by the action of thromboxane A2 synthase on prostaglandin H2 (PGH2) which is a byproduct of cyclo-oxygenase-2 (COX-2) and AA [55]. Whereas, non-enzymatically, MDA is reported to be produced by the formation of bicyclic endo-peroxides during lipid peroxidation under both stressed or non-stressed conditions [56].

It is a well known phenomenon that the stimulus for the inflammation is triggered due to alveolar hypoxia, which is initially localized and then becomes systematic [57]. Many of the earlier studies have also clearly stated the association of inflammation and increased ROS production at mitochondrial level in causing the fluid build-up in the alveolar spaces [58]. Literature has also suggested that hypoxia contributing to inflammation has gained greater acceptance as it is reported to be responsible for hypoxia signalling cascades [59].Members of the family of nuclear factor ĸB (NFĸB) gets amplified and interacts with the members of PHD-HIF family at low PO_2_ levels [60,61]. In the present study, theNFĸB was increased under hypoxia followed by elevated HIF-1α expression compared to normoxia control (0h).

Based on the above discussed interaction, the production of pro-inflammatory cytokines such as-TNF-α, INF-γ etc. up regulated under hypoxia[62]. Studies bySemenza G.L. (2007) and Grocott et.al., (2009) have also confirmed the elevated levels of pro-inflammatory cytokines and leakage of vascular fluid in lungs of mountaineers at high altitude [63,64]. Moreover, the studies on mice exposed to reduced oxygen concentration for shorter duration have also resulted into the enhanced levels of cytokines in serum, accumulation of pro-inflammatory cells in various tissues and vascular leakage [65,66]. In addition to this, IĸB- complex, which is a well known regulator of NF- ĸB also aggravates inflammation under hypoxic condition by enabling the transcription of HIF-1α mediated by NF- ĸB activation[67]. Another interesting finding in this study was that quercetin (50 mg/kg BW) efficiently inhibited the NFĸB mediated cell adhesion molecules (ICAM-1, VCAM-1 and P-selectin) present in lungs of the rats under hypoxia. In normal rat lung, moderately low levels of intercellular cell adhesion molecules-1 (ICAM-1) are reported to be constitutively expressed in the capillary endothelium cells and on the surface of type-I pneumocytes [68]. These studies have also reported that the factors stimulating pro-inflammatory cytokines essentially- TNF- α, IL-1, IL-6 etc., triggers the expression of ICAM-1 in lung microvasculature [69]. Similarly the vascular cell adhesion molecule-1 (VCAM-1) a well known adhesion molecule found prominently in lung vascular tissues, is importantly recognized by its antigen-induced recruitment of eosinophils and T cells [70]. In lung microvasculature VCAM-1 expression is reported to be regulated by inflammatory cytokines especially- TNF- α and IL-1 or variably by LPS [71]. Studies on VCAM-1 have also evidenced under hypoxic condition, hypoxia induced mitogenic factor (HIMF) responsibly promotes the VCAM-1 overexpression via NF- ĸBsignalling[72]. In addition to this, P-selectin, another cell adhesion molecule lining the activated platelets and blood vessels, present all over the surface of activated endothelial cells plays pivotal role in influx of polymorphonuclear neutrophils (PMNs) in various inflammatory conditions especially at low oxygen concentration due to NFĸB signaling stimulation[72].However, at molecular level, quercetin prophylaxis has demonstrated the efficient reduction in inflammation by attenuating pro-inflammatory cytokines production mediated by the reduced expression of IKKα/β, NFĸB, and cell adhesion molecules along with the dramatic increase in anti-inflammatory cytokines expressions along with stabilized HIF-1α. This perhaps leads to the reduced vascular leakage in hypoxia exposed animals under quercetin prophylaxis as evidenced by reduced albumin and LDH levels in lungs of rats.The attenuation of inflammation (NFkB) and the oxygen homeostasis (HIF-1α) is found to be the potent anti-inflammatory and strong anti-oxidant activity of quercetin. It indicates that quercetin is a potent molecule to reduce inflammation and oxidative stress under reduced oxygen conditions.

Previous studies have documented that, out of two mountain climbers one develops the signs and symptoms of HAPE with the drastic changes in lung histology [73]. Similar reports were also presented in studies carried out on lung tissues of rats exposed to reducing environment, which demonstrated the fluid build-up, alveolar thickening and pulmonary edema in the lungs [74,75]. Simultaneously, the biochemical findings were further confirmed by histopathological examination of lung sections of control and hypoxia exposed animals. The results exhibited the normalization of hypoxia induced changes in lung configuration upon quercetin prophylaxis.

Therefore, it can be interpreted that quercetin prophylaxis at molecular level attenuates hypoxia induced transvascular leakage by down regulating the inflammation, alveolar oxidative stress, extravasation of plasma proteins into the lung interstitium, promoting the production of anti-inflammatory cytokines and stabilizing the expressions of HIF-1α followed by reduced VEGF expressions resulting into the prevention of hypoxia induced pulmonary edema. All these findings clearly evidence the potential use of quercetin in clinical applications.

## Conclusion

The present study reveals that,the quercetin (50 mg/kg BW) prophylaxis under hypoxia was found to be effective in minimizing the severity of HAPE by reducing the fluid flux into the lungs of rat model, thus it may provide similar protection in humans against HAPE. Therefore, the conclusion drawn from the present study was that, the prophylactic administration of quercetin abrogates the possibility of hypobaric hypoxia induced pulmonary edema in rats.

## Supporting information

S1 File Effect of hypoxia on ROS. (XLSX)

S2 File Effect of hypoxia on MDA. (XLSX)

S3 File Measurement of GSH under hypoxic condition. (XLSX)

S4 File Determination of edema index under hypoxic environment. (XLSX)

S5 File Determination of transvascular leakage in the lungs of rats under hypoxia. (XLSX)

## Abbreviations

A: Alveoli
AA: Arachidonic Acid
AS: Alveolar Spaces
BV: Blood vessel
COX-2: Cyclo-oxygenase-2
DTT: Dithiothreitol
HAPE: High Altitude Pulmonary Edema
HH: Hypobaric Hypoxia
Hif-1α: Hypoxia Inducible Factor-1α
His-H3: Histone-H3
4-HNE: 4- Hydroxynonenal
IL: Interleukin
IFN-γ: Interferron- γ
ICAM: Intercellular Adhesion Molecule
IKK: Inhibitor of nuclear factor Kappa-B Kinase.
LDH: Lactate Dehydrogenase
MDA: Malondialdehyde
Nrf-2: Nuclear factor erythroid 2-related factor-2
NF κ B: Nuclear factor κ- light chain enhancer of activated B-cells
PGH2: Prastaglandin H2
PMSF: Phenyl Methyl Sulfonyl Fluoride
PIC: Protease Inhibitor Cocktail
ROS: Reactive oxygen species
RT: Room Temperature
SD: Sprague Dawley
SOD: Superoxide dismutase
TNF-α: Tumor Necrosis Factor- α
TGF-β: Tumour Growth Factor-β
TBA: Thio Barbituric Acid
TCA: Tri Carboxylic Acid
VEGF: Vascular Endothelial Growth Factor
VCAM: Vascular Cell Adhesion Molecule
